# Neuroendocrine Differentiation Is a Prognostic Factor for Stage II Poorly Differentiated Colorectal Cancer

**DOI:** 10.1155/2014/789575

**Published:** 2014-06-29

**Authors:** Yue Liu, Jinghong Xu, Yurong Jiao, Yeting Hu, Chenghao Yi, Qiong Li, Zhou Tong, Xiaowei Wang, Lifeng Hu, Qian Xiao, Jun Li, Kefeng Ding

**Affiliations:** ^1^Cancer Institute, Key Laboratory of Cancer Prevention and Intervention, China National Ministry of Education and Key Laboratory of Molecular Biology in Medical Sciences, The Second Affiliated Hospital, School of Medicine, Zhejiang University, 88 Jiefang Road, Hangzhou, Zhejiang 310009, China; ^2^Department of Pathology, The Second Affiliated Hospital, School of Medicine, Zhejiang University, 88 Jiefang Road, Hangzhou, Zhejiang 310009, China; ^3^Department of Surgical Oncology, The Second Affiliated Hospital, School of Medicine, Zhejiang University, 88 Jiefang Road, Hangzhou, Zhejiang 310009, China

## Abstract

Neuroendocrine differentiation (NED) in colorectal cancer is an indistinct phenomenon and may define a new cancer subtype, especially in the poorly differentiated colorectal cancer (PDCRC). The clinical features of PDCRC with NED remain controversial, thus confusing the implementation of individualized treatment. This study included 171 patients who underwent surgery from 2000 to 2011 and had pathology-confirmed PDCRC. Each sample was examined by immunohistochemistry for the biological markers of NED, synaptophysin (Syn), and chromogranin (CgA). Patients with Syn(+) and/or CgA(+) cells were classified as NED(+); otherwise, they were NED(−). Data were collected for patients who were followed up for at least two years. NED(+) staining was present in 71 (41.5%) patients. The median survival time was 36.9 months. No survival differences existed between the NED(−) and NED(+) groups (*P* > 0.05). However, stage II NED(+) patients had a significantly worse prognosis than NED(−) patients (*P* = 0.018). For the NED(+) group, the median survival was 38.56 months, and the 5-year survival was 65%. For the NED(−) group, the median survival was 53.18 months, and the 5-year survival was 90%. NED is a common event in primary PDCRC. For stage II PDCRC, NED(+) indicates a poor prognosis.

## 1. Introduction

Poorly differentiated colorectal cancer (PDCRC) composes 4.8% to 20% of all colorectal cancers which is usually associated with a poor prognosis [1–3]. The diagnosis of PDCRC with neuroendocrine differentiation (NED) depends mainly on the commonly used neuroendocrine (NE) markers synaptophysin (Syn) and chromogranin A (CgA). However, the clinicopathological features and prognostic significance of NED in PDCRC remain unclear.

According to previous studies, the incidence of NED in colorectal cancer varies significantly, ranging from 11.9% to 77.5% [4–10]. The wide range of values could be attributed to data obtained from different races and geographical regions, but importantly it could also be due to different diagnostic markers and different diagnostic standards. As immunohistochemistry (IHC) is the main method to diagnose NED, different IHC biomarkers are very important. Different sensitivities and specificities for each marker, such as CgA, Syn, CD56, NSE, syntaxin1, VAMP2, SNAP25, and *α*/*β*-SNAP, may cause significant variation in the reported incidence of NED. Additionally, the definition of NED still lacks a quantitative standard, and thus different study centers may use different standards [5, 11–13].

Conflicting data exists in previous studies evaluating the clinical prognostic value of NED for colorectal cancer (CRC). In a study by Grabowski et al. [5] of 146 patients with stages III and IV colorectal cancer, NED was associated with a poor prognosis. Similarly, Gulubova and Vlaykova [6] showed that, in 137 CRC patients, NED was a useful marker for a poor prognosis after surgical therapy regardless of the tumor, node, metastasis (TNM) staging. On the other hand, Foley et al. [7] studied 48 stage III patients and found no prognostic value for NED in CRC patients. Similar findings were reported by Cho et al. [8] for 89 stage II CRC patients. Intriguingly, some studies indicated that CRC with NED was correlated with liver metastasis and advanced stages [9, 10].

In this study, we evaluated the prognostic value of NED in patients with PDCRC. We analyzed the most commonly used IHC NE markers Syn and CgA to determine the relationship between clinicopathological manifestation and neuroendocrine differentiation in PDCRC.

## 2. Materials and Methods

Between 2000 and 2011, all 177 patients who received primary colorectal cancer resection and who were confirmed by pathology to have poorly differentiated adenocarcinoma were analyzed retrospectively. Patients with perioperative mortality (survival shorter than 3 months after the operation) and with secondary malignancy were excluded.

## 3. Immunohistochemistry

Paraffin-embedded tissue sections (4 *μ*m) were immunostained by a two-step method. After deparaffinization and antigen retrieval, endogenous peroxidase activity and nonspecific antigen binding sites were blocked by successive incubation with 3% hydrogen peroxide for 10 minutes and 5% BSA for 30 minutes. Then the tissue sections were incubated with CgA rabbit monoclonal antibody (AC-0037, dilution 1 : 200, Epitomics, Burlingame, CA, USA) or Syn rabbit monoclonal antibody (AC-0163RUOBULK, dilution 1 : 200, Epitomics) at 37°C for 2 hours. Bound antibodies were detected by applying Dako REAL Envision Detection System (peroxidase/DAB+, rabbit/mouse, K5007, Dako, Glostrup, Denmark), and the sections were counterstained with Mayer's hematoxylin. Negative controls were prepared by following the same procedure but omitting the primary antibody.

**Figure 1:**
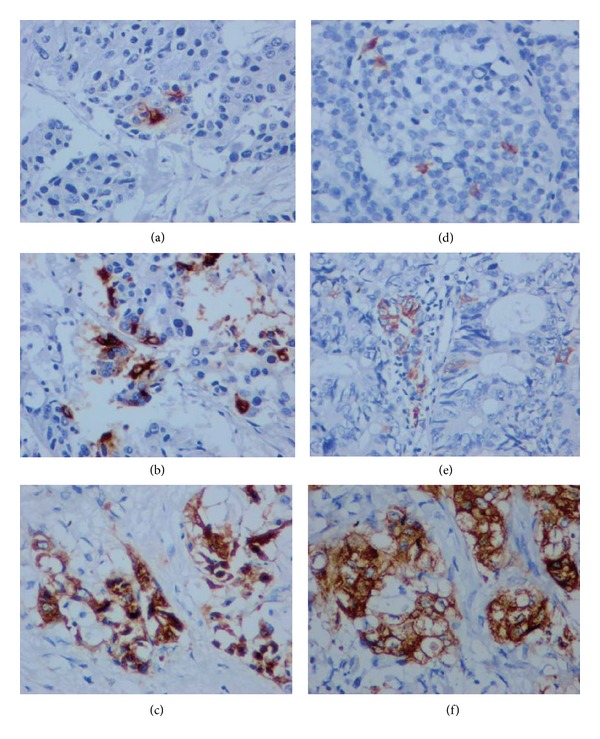
IHC of CgA ((a), (b), and (c)) and Syn ((d), (e), and (f)) (400x). Subgroup 1, (a) and (d), scattered stained, 1–10 NED(+) tumor cells/HPF; subgroup 2, (b) and (e), focally stained, 11–20 NED(+) tumor cells/HPF; subgroup 3, (c) and (f), clustered or pathy stained, >20 NED(+) tumor cells/HPF.

The slides were evaluated by 2 senior pathologists. The presence of CgA- and Syn-immunoreactive cells was evaluated in all tumor fields. When no immunoreactive tumor cells for CgA and Syn were noted, the tumor was designated as NED(−). When ≥1 tumor cells/HPF (high power field) were positive for CgA and/or Syn, the tumor was designated as NED(+). Furthermore, the 10 most concentrated areas of NED(+) cells at ×400 magnification were evaluated, and the results were presented as the mean number of Syn- and/or CgA-positive cells per HPF. Tumors were assigned to subgroups based upon the number of Syn- and/or CgA-positive cells per HPF: subgroup 1 had 1–10 Syn- and/or CgA-positive cells/HPF; subgroup 2 had 11–20 Syn- and/or CgA-positive cells/HPF; subgroup 3 had more than 20 CgA- and/or Syn-positive cells/HPF (Figure 1).

### 3.1. Clinical Data

The clinicopathological data were collected from the history case records retrospectively. Survival time was measured as the last follow-up date minus the operation date for living patients and the date of death minus the operation date for deceased patients.

### 3.2. Statistical Analysis

Statistical analyses were performed with SPSS (version 18.0). Correlation between NED(+) and NED(−) groups and other variables were investigated by Pearson's chi-squared test. Overall survival was assessed by the Kaplan-Meier method. The statistical significance of differences between the survival curves was calculated by the log rank test. Significant variables were then examined by multivariate analysis using the Cox model to generate hazard ratios (HR) and 95% confidence intervals (95% CI). Values with *P* < 0.05 were considered to be significantly different.

## 4. Results

### 4.1. General Information

Based on Syn(+) or CgA(+) staining, six stage III patients were diagnosed as neuroendocrine tumor/neuroendocrine carcinoma (NET/NEC) or mixed adenoneuroendocrine carcinoma (MANEC) according to the 2010 World Health Organization (WHO) classification. These patients were excluded from further study.

There were 171 patients (98 males and 73 females), including 115 cases of colon cancer and 56 cases of rectal cancer. Among them, 8 had stage I cancer, 42 had stage II, 93 had stage III, and 28 had stage IV. The mean ± standard deviation age was 60.3 ± 14.3 years (range: 19–89 years). Eighty-five patients received adjuvant chemotherapy after surgery, and the median survival time was 36.9 months. Sixty-nine of the 171 patients died during the follow-up period, including 2 of the 8 patients in stage I, 7 of the 42 in stage II, 37 of the 93 in stage III, and 23 of the 28 in stage IV.

### 4.2. Clinicopathological Parameters and Neuroendocrine Markers

NED(+) cells were detected in 71 cases (41.5%). For cancer stages I, II, III, and IV, the proportion was 37.5%, 33.33%, 49.46%, and 28.57%, respectively. There were no significant correlations between NED and age, lymphatic invasion, TNM stage, or discovered lymph nodes (*P* > 0.05, Table 1). However, females were more prone to have NED(+) cells than males (*P* = 0.019, Table 1). There were 71 NED(+) cases with the clinicopathological features shown in Table 1. Of these, 29 cases were in subgroup 1, 11 cases were in subgroup 2, and 31 cases were in subgroup 3.

### 4.3. Prognostic Implication of Neuroendocrine Differentiation

**Figure 2:**
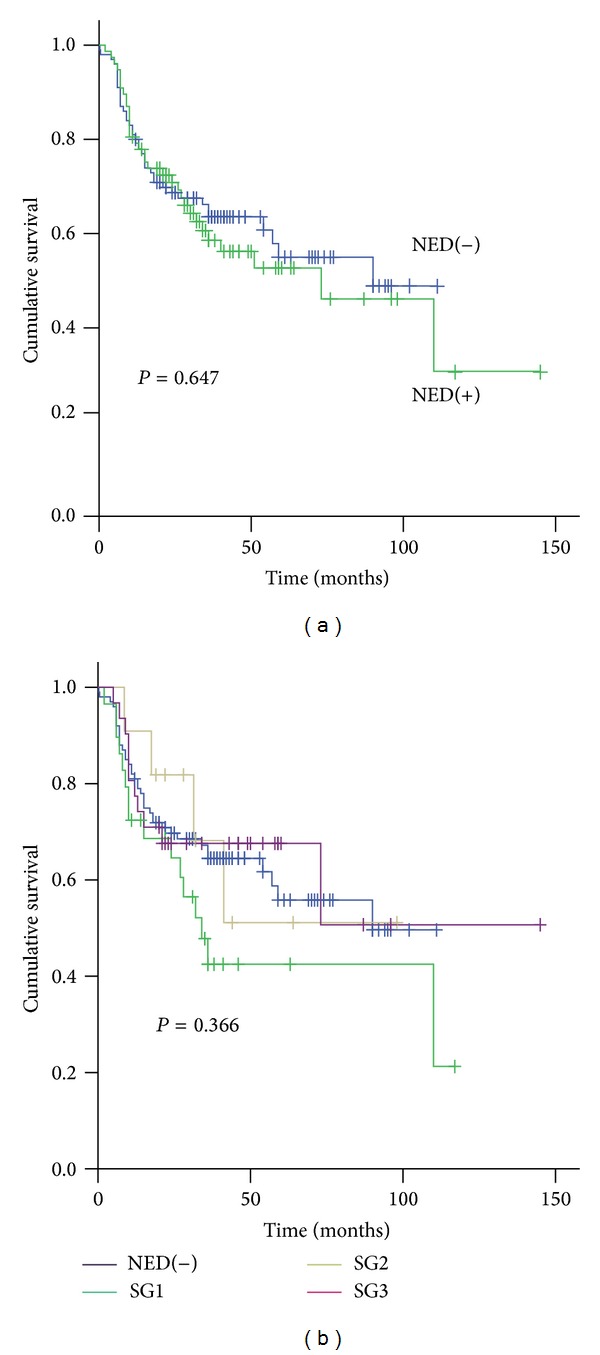
Prognostic relevance of NED in PDCRC. (a) Kaplan-Meier survival analysis of all patients. There were no survival differences between the NED(+) and NED(−) groups. (b) The NED(+) group was divided into subgroups (SG) 1, 2, and 3 according to the number of NED(+) cells per HPF. While none of the 3 subgroups were significantly correlated with cumulative survival, subgroup 1 tended to have a worse prognosis than did the NED(−) group.

For all of the PDCRC cases, there was no significant difference in survival between the NED(+) group and the NED(−) group. However, the NED(+) group tended to have a worse prognosis than did the NED(−) group (*P* = 0.647, Figure 2(a)). We also analyzed the prognosis associated with NED(+) subgroups 1, 2, and 3. There was no correlation between the subgroups and the cumulative survival in PDCRC (*P* = 0.366, Figure 2(b)). Interestingly, the NED(−) group tended to have a better prognosis than did the NED(+) subgroup 1 (*P* = 0.119), but the difference was smaller between the NED(−) group and NED(+) subgroup 2 (*P* = 0.882) and NED(+) subgroup 3 (*P* = 0.829).

**Figure 3:**
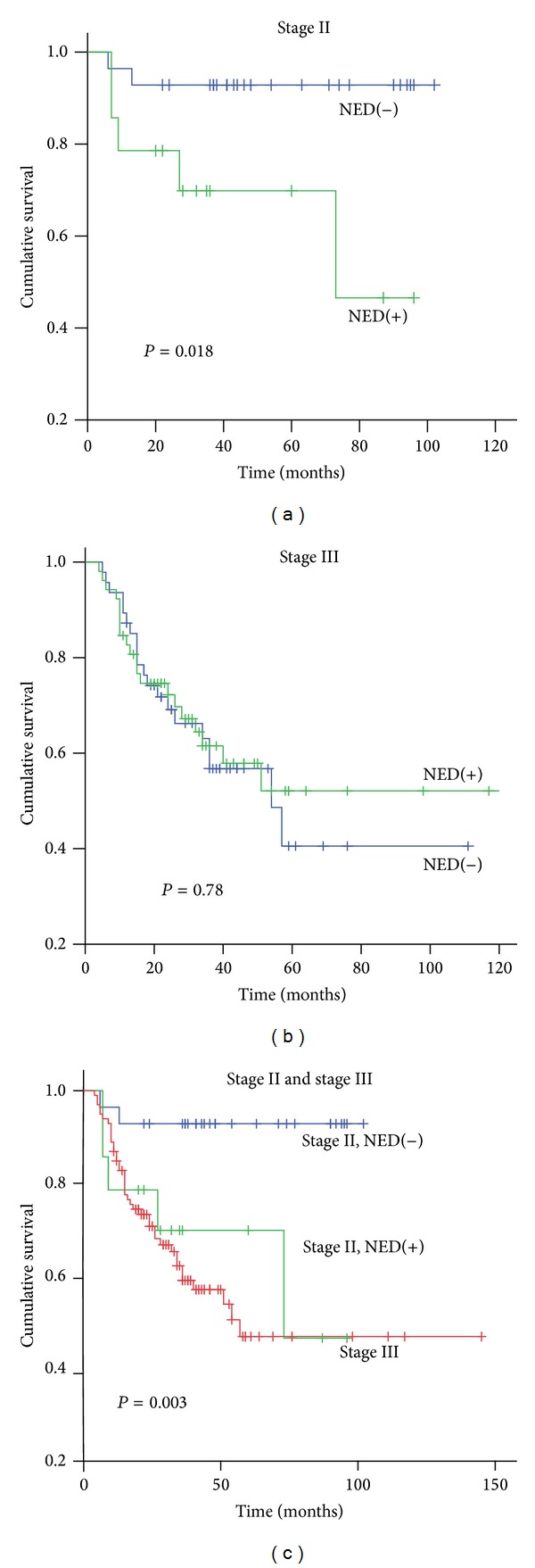
Kaplan-Meier survival analysis by stratification of TNM staging. (a) In stage II, the NED(+) group had a significantly worse prognosis than the NED(−) group (*P* = 0.018). (b) In stage III, there was no significant difference in survival time between the NED(+) and NED(−) groups. (c) There was no significant difference in the survival times of stage II NED(+) and stage III patients (*P* = 0.898). However, the survival time of stage II NED(−) patients was significantly longer than stage II NED(+) patients (*P* = 0.008) and stage III patients (*P* = 0.001).

By stratification analysis based on TNM staging, for patients with stage II PDCRC, the NED(+) group had a worse prognosis than did the NED(−) group (log rank, *P* = 0.018; Figure 3(a)). In the NED(+) group, the median survival was 38.56 months (range: 7 to 87 months), and the 5-year survival rate was 65%. In the NED(−) group, the median survival was 53.18 months (range: 6 to 102 months), and the 5-year survival rate was 90%. For stage III, the overall survival was not different between the two groups (log rank, *P* = 0.78, Figure 3(b)). We then compared the overall survival of stage II patients with NED(+) or NED(−) to stage III patients. Stage II patients with NED(−) had a significantly better prognosis than stage II patients with NED(+) or stage III (*P* = 0.003, Figure 3(c)). There was no significant difference between stage II patients with NED(+) and stage III patients (*P* = 0.656).

**Figure 4:**
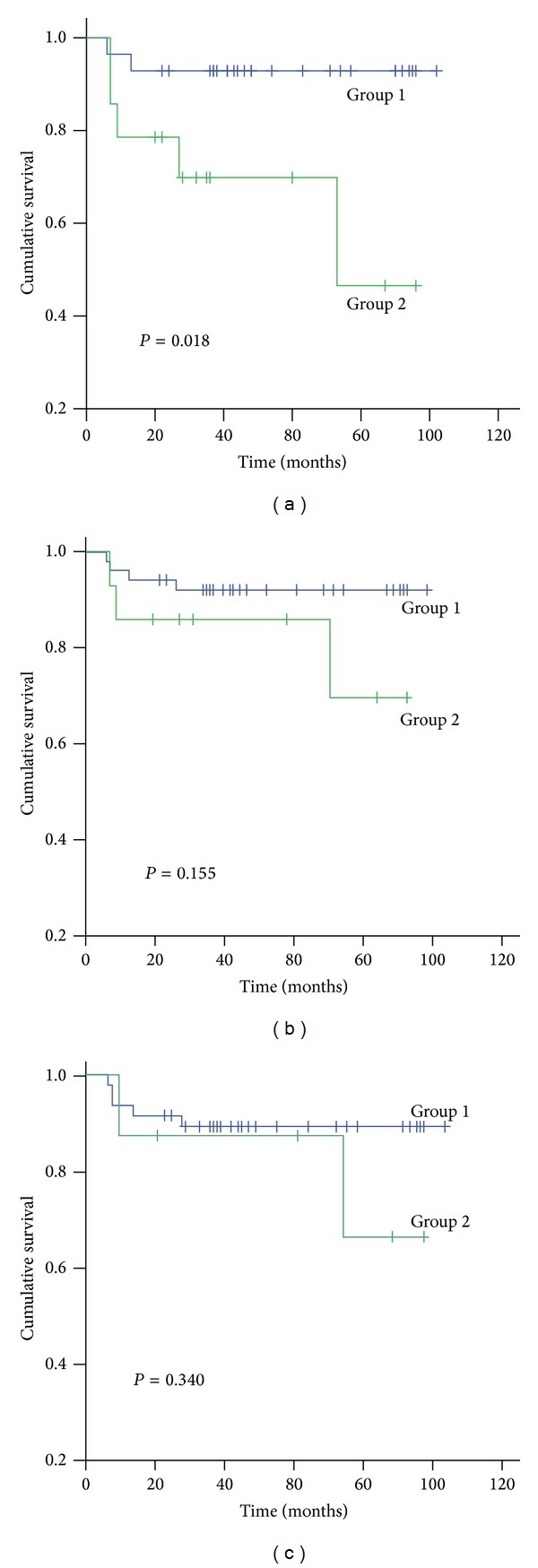
Effect of different cutoff values on cumulative survival rate for stage II PDCRC patients. Group 1 refers to the cases with Syn- and/or CgA-positive cells below the cutoff value while Group 2 refers to the cases above the cutoff value. (a) Kaplan-Meier survival analysis showed that, for a cutoff value of one Syn- and/or CgA-positive tumor cell/HPF, the survival of Group 1 patients was greater than that of Group 2 patients (*P* = 0.018). When the cutoff value was 10 (b) or 20 (c) Syn- and/or CgA-positive tumor cells/HPF, there was no survival difference between the groups (*P* = 0.155 and *P* = 0.340, resp.).

Stage II PDCRC was further separated into only two groups by using different cutoff values (Figure 4). Stage II Group 1 refers to the cases with Syn- and/or CgA-positive cells below the cutoff value while stage II Group 2 refers to the cases with Syn- and/or CgA-positive cells above the cutoff value. With cutoff values of 10 (Figure 4(b)) or 20 (Figure 4(c)) Syn- and/or CgA-positive tumor cells per HPF, Kaplan-Meier survival analysis showed that there was no survival difference between stage II Group 1 and Group 2 (*P* = 0.155, 0.340, resp.), while, with a cutoff value of 1 Syn- and/or CgA-positive cell per HPF (Figure 4(a)), the survival difference existed (*P* = 0.018).

The clinical and pathological variables, including age, sex, tumor location, discovered lymph nodes, T stage, chemotherapy receipt, lymphatic invasion, adjuvant chemotherapy, and NED, were first analyzed by univariate analysis on overall survival in stage II PDCRC. Among these, only T stage (*P* = 0.003), NED (*P* = 0.018), and age (*P* = 0.019) were significant (Table 2). To assess independent prognostic information among the variables, multivariate Cox analysis was used to generate a model. The only independent prognostic factors (Table 3) were T4 stage (HR, 6.084; 95% CI, 1.732–21.364; *P* < 0.01) and NED(+) (HR, 7.700; 95% CI, 1.397–42.446; *P* < 0.05). No significant correlation was present between NED and any of the other clinical and pathological variables in stage II PDCRC (Table 2).

## 5. Discussion

NED occurs in a variety of cancers, including colorectal cancer. Our understanding of it is based on ultrastructural and/or immunohistochemical studies [4] that rely on immunohistochemical markers [14]. However, the selection of markers used in the diagnosis of NED has been problematical. NE markers such as CgA, Syn, NSE, CD56, serotonin, and others have been used. CgA and Syn are the most frequently used markers of NE cells in colorectal cancer studies [4, 14, 15]. Each marker is unique and localized in separate intracellular granules, and one or the other or both may be present. CgA is found in cells with large dense-cored vesicles (LDCV), and Syn is present in small synaptic vesicles (SSV). According to Schmitt-Gräff et al., in high-grade malignant carcinomas, the reduction of LDCV matrix protein CgA was highly significant, while, in contrast, the amount of Syn appeared to be better maintained [16]. Grabowski et al. [4] reported on 45 undifferentiated colon cancers in which CgA, Syn, syntaxin1, VAMP2, SNAP25, and *α*/*β*-SNAP were used as NE markers. Nine cases were diagnosed as NED(+). Among those cases, CgA was expressed in only 5 tumors, whereas all 9 tumors expressed Syn. Another study also implied that not all CgA(+) tumor cells were also Syn(+) [6]. In our study, the rate of CgA(+) was 15.8% while that of Syn(+) was 35%. Eleven tumors expressed both Syn(+) and CgA(+). Because CgA and Syn are present in separate NE granules, assessing both of these markers is essential for the detection of NE cells. For diagnostic purposes, it would be inadequate to look for only one of these markers of NED.

Another problem in the selection of NE markers is the cutoff value in the definition of NED. Two main cutoff values have been used. In a study by de Bruine et al., the counting of CgA(+) tumor cells per mm^2^ of tumor surface area was done at a magnification of 312.5x [11]. In their analysis, the absence of observed CgA(+) cells was recorded as “negative.” When the number of CgA(+) cells was less than 1 per mm^2^ in the section, it was designated as “moderate positivity,” and when the number exceeded 1 per mm^2^, it was designated as “extensive positivity.” In that study of 350 cases, CgA(+) tumor cells were found in 30% of the cases, with 21% showing moderate positivity and 9.0% having extensive positivity. Survival for adenocarcinoma with NED tended to be poorer than for non-NE tumors (*P* = 0.068).

The other cutoff value was established by Grabowski et al. [5]. They counted immunoreactive tumor cells in at least three tumor fields. Neoplasms showing very few scattered immunoreactive cells were scored as negative (nonexpressers). Positive tumors were classified as “low expressers” when there were less than 2% immunoreactive cells and as “high expressers” when there were more than 2% immunoreactive cells. In that study of 116 cases containing stage III and stage IV cancers, the rate of nonexpressers, low expressers, and high expressers was 63.9%, 18.9%, and 17.2%. In the survival analysis, the presence of more than 2% of cells with NED was found to be an independent prognostic parameter for stages III and IV disease. Other cutoff values have also been used, such as 10% (12) and 5 NED(+) cells/mm^2^ [13].

There are some differences in the NE neoplasm classification system used today. According to the WHO classification of gastroenteropancreatic neuroendocrine tumor (GEP-NET) [17], when the NE component and adenocarcinoma component are each ≥30%, it is classified as a MANEC. In previous studies, the “extensive positivity” or “high expressers” included MANEC, and they may have even contained neuroendocrine carcinoma (NEC), which has a poor prognosis. Six out of our 177 patients were diagnosed with NEC or MANEC, and they were primarily classified as stage III. This indicates that, in the previous category system, MANECs were not separated from the adenocarcinomas. Using the current definition of NED, this may influence the incidence and prognostic role, so reevaluation of NED is necessary.

We used CgA and Syn as NE markers to try to set a scale to evaluate NED. Grabowski et al. [4] defined “low expression” as less than 2% of the tumor cell population expressing CgA or Syn or both because the normal colorectal epithelium may contain up to 2% NE cells. So we initially defined the NED(−) tumors as having <2% CgA(+) or Syn(+) cells. The NED(+) rate was just 11.11%, and there was no survival difference between NED(+) and NED(−) groups (*P* = 0.692). In our observation, tumor cells positive for CgA, Syn, or both were scattered or focally clustered in most of the tumors. Additionally, the CgA(+) and Syn(+) tumor cells were distributed differently from the NE cells in the normal mucosa. This is in accordance with Gulubova and Vlaykova [6], who reported 0.257 CgA(+) cells/mm^2^ in the tumor. In the normal mucosa, they found that NE cells were located mainly at the base of the glands and contained pleomorphic or oval electron dense granules located beneath the spherical nucleus. In contrast, the tumor NE cells were located at the periphery of the glands and had nuclei that were indented. Further, the CgA(+) and Syn(+) granules were dispersed throughout the entire cytoplasm and were structurally pleomorphic with membranes that were tightly applied to the dense core.

In our study, we defined NED(+) tumors as those having ≥1 immunoreactive tumor cell/HPF, which is similar to the criteria used by Gulubova and Vlaykova [6] and Eschrich et al. [18]. The incidence of NED in PDCRC was 41.5%, and the overall survival was not significantly different between NED(+) and NED(−) groups for all 171 PDCRC tumors. However, the NED(+) group tended to have a worse prognosis. Furthermore, we divided the NED(+) cases into 3 subgroups according to the number of positive NED(+) cells. The survival curve showed that subgroup 1 contributed more to the poor prognosis of the NED(+) group than did subgroups 2 and 3. This indicated that even a small number of positive NED(+) cells may influence the survival of PDCRC.

To refine the analysis of stage II PDCRC, we separated the cases into only two groups by using different cutoff values. Kaplan-Meier survival analysis showed that there was no survival difference between stage II Group 1 and Group 2 with cutoff values of 10 or 20 Syn- and/or CgA-positive tumor cells per HPF, respectively. However, with a cutoff value of 1 Syn- and/or CgA-positive cell per HPF, the survival rate of the stage II Group 1 patients was worse than the stage II Group 2 patients. These data indicate that it is reasonable to set the cutoff value of NED(+) cells at a low level.

In our study, the NED(+) group presented a worse prognosis than the NED(−) group in stage II PDCRC, while, for all the PDCRC cases, the survival difference was not significant. In contrast, Gulubova and Vlaykova reported that, in all the CRC patients, the overall survival for NED(+) patients was worse than for NED(−) patients [6]. Interestingly, the difference in both of our findings may be partially attributed to the TNM stage proportion of the samples. In Gulubova and Vlaykova's study, stage II composed 58.6% of the tumors, while stage III was 14.3%. In contrast, our sample sizes were 24.6% stage II and 54.3% stage III. Gulubova and Vlaykova also showed that the relationship between NED status and survival was even more pronounced when only the patients with less advanced tumors (stages I or II) were analyzed.

TNM staging is a classic staging method to predict survival and determine the administration of adjuvant chemotherapy. However TNM staging for stages II and III patients is less reliable in predicting survival. Eschrich et al. [18] argued that molecular staging may provide an accurate prognostic value for patients. According to them, stages II and III patients should be further subdivided into good and poor prognosis groups in which the survival of the stage II poor prognosis group is worse than the stage III good prognosis group. Based on the traditional clinical stage classification, stages III and IV patients should receive chemotherapy. However, this now is being challenged by the molecular staging method, which recommends that the stage II patients with poor prognosis should also receive adjuvant chemotherapy. According to the 2013 Colorectal Cancer National Comprehensive Cancer Network (NCCN), the high risk stage II patients should receive adjuvant chemotherapy. These risk factors include <12 lymph nodes discovered after surgery, poor prognostic features (poorly differentiated histology), lymphatic/vascular invasion, bowel obstruction, perineural invasion, localized perforation, and close or positive margin.

Finding important molecular markers with prognostic significance has become increasingly important. We found that, in stage II PDCRC, the NED(+) group had a much worse prognosis than the NED(−) group, and the survival time of stage II NED(+) group was similar to that of the stage III group. Therefore, our data strongly indicate that the NED(+) group is an important subtype in stage II PDCRC. This subtype represents a poor prognosis, for which the administration of adjuvant chemotherapy is probably needed. However, adjuvant chemotherapy was not correlated with NED in our study. This may be in part due to the small sample size and few patients who received chemotherapy. This merits further study.

In conclusion, neuroendocrine differentiation is a common event in primary poorly differentiated colorectal cancer. For stage II PDCRC patients, NED is a poor prognostic factor.

## Figures and Tables

**Table 1:** Correlation between NED and clinicopathological parameters.

Property	NED		*P* value
	Negative	Positive total (SG1, SG2, and SG3)	
Age			
<65	59	38 (14, 5, 19)	0.532
≥65	41	33 (15, 6, 12)	
Gender			
Male	65	33 (10, 6, 17)	0.019
Female	35	38 (19, 5, 14)	
Tumor location			
Colon	70	45 (14, 8, 23)	0.410
Rectum	30	26 (15, 3, 8)	
Lymphatic invasion			
Yes	24	19 (9, 1, 9)	0.722
No	76	52 (20, 10, 22)	
Lymph nodes discovered			
≥12	70	53 (22, 8, 23)	0.605
<12	30	18 (7, 3, 8)	
TNM stage			
I	5	3 (1, 1, 1)	0.135
II	28	14 (5, 3, 6)	
III	47	46 (18, 6, 21)	
IV	20	8 (5, 1, 3)	
Chemotherapy			
Yes	54	28 (11, 4, 13)	0.065
No	46	43 (18, 7, 18)	

SG: subgroup; SG 1: 1–10 positive cells/HPF; SG 2: 11–20 cells/HPF; SG 3: >20 cells/HPF; NED: neuroendocrine differentiation; TNM: tumor, node, metastasis.

**Table 2:** Univariate analysis of clinicopathological factors for overall survival in stage II PDCRC.

Factor	*N*		Kaplan-Meier log rank test *P* value
	NED(−)	NED(+)	
Age			
<65	15	3	0.019
≥65	13	11	
Gender			
Male	15	3	0.391
Female	13	11	
Primary tumor location			
Colon	24	10	0.174
Rectum	4	4	
LND			
<12	8	7	0.224
≥12	20	7	
Chemotherapy receipt			
Yes	11	4	0.809
No	17	10	
T stage			
T3	15	8	0.003
T4a	10	5	
T4b	3	1	
Lymphatic invasion			
Yes	2	2	0.469
No	26	12	
NED			
Positive	/	14	0.018
Negative	28	/	

LND: lymph node discovered; NED: neuroendocrine differentiation.

**Table 3:** Independent prognostic factors in multivariate analysis for overall survival in stage II colorectal cancer.

Factor	Overall survival			
	*N*	HR	95% CI	*P* value
T4 stage	42	6.084	1.732–21.364	0.005
NED(+)	42	7.700	1.397–42.446	0.019
